# Energy consumption, CO_2_ emissions and electricity costs of lighting for commercial buildings in Southeast Asia

**DOI:** 10.1038/s41598-022-18003-3

**Published:** 2022-08-13

**Authors:** Wan Yun Hong, Bibi Nurmuslihah Ni’matullah Nura’liyah Rahmat

**Affiliations:** 1 Faculty of Integrated Technologies, Universiti Brunei Darussalam, Jalan Tungku Link, Gadong, BE1410 Brunei Darussalam; 2 Present Address: Muara Maritime Services, Muara Naval Base, Jalan Tanjong Pelumpong, Serasa, BT1328 Brunei Darussalam

**Keywords:** Energy economics, Energy efficiency, Climate-change mitigation

## Abstract

Building energy intensity (BEI) has been used to assess a building’s overall energy performance. However, the energy performance, CO_2_ footprint and electricity costs due to lighting in buildings are currently required to assist relevant authorities to develop, revise and implement energy-efficient lighting policies that are effective and acceptable for the country. This work presents an estimation approach for lighting in commercial buildings in Southeast Asia and its decarbonisation pathway for benchmarking. Application of this approach to a selected library in Brunei Darussalam showed that an energy-efficient light-emitting diode (LED) lighting system would make the building greener. We projected reductions in lighting energy consumption by 6.7 times (3.98 kWh/m^2^/year), its associated CO_2_ emissions by 8 times (0.59 kg CO_2_/m^2^/year) and electricity costs by 8.7 times (B$7.07/m^2^/year) by 2050 if existing lamps in the library are retrofitted with LED lamps.

## Introduction

According to a report by the United Nations Environment Programme (UNEP) in 2013, almost 20% of electricity consumption and 6% of carbon dioxide (CO_2_) emissions worldwide were attributed to electricity for lighting^1^. Lighting is one of the major end-use of electricity that accounts for approximately 48% of the building electricity in the commercial sector and 28% of the building electricity in the residential sector globally in 1997^2^. Due to the long operating hours and a large number of lamps installed in the commercial sector, its electrical energy demand for lighting tends to be higher than the residential sector^3^. Without a rapid change in policies and practical implementations to transition to energy-efficient lighting such as light-emitting diodes (LEDs), the global energy consumption for lighting is expected to rise by 60% by 2030 and this will also increase the energy-related CO_2_ emissions, thus causing more warming of the Earth and further climate changes in the future^1,4^.

The global energy efficiency policy coverage for buildings continued to expand to about 35% of total energy consumption covered by policies in 2018 despite having a slower annual growth rate of 2% to 3% in recent years^5^. The major efforts to phase out incandescent lamps since 2008 by many countries around the world have raised policy coverage for lighting but the annual energy policy improvements for lighting in 2017 and 2018 were insignificant and it seems to be saturated at about 83% of total energy consumption covered by policies in 2018^5,6^. Many policies need to be revised and updated to raise energy efficiency standards and include product labelling requirements^7^. Policies need to include more regulatory and financial incentives (such as tax incentives, import duties, subsidies, rebates, giveaways, incentives for manufacturers/suppliers and vendor incentives) to make low-carbon and energy-efficient products (such as LEDs) more affordable to all, which can help increase public acceptance of efficient lighting strategies^5,7^. Nearly 29% of the countries in the world are making progress by adopting lighting policies for transitioning to efficient lighting and nearly 58% of the countries in the world have not yet established energy-efficient lighting policies, which may delay the global ambition to reach net-zero emissions by 2050^7^.

LED deployment in buildings has progressed steadily with over 50% of global lighting sales in 2020 and this share will need to raise to 100% globally by 2025 in order to meet the net-zero emissions target by 2050^8,9^. LED lamps are now the global leading lighting technology as many countries have started to phase-out fluorescent and halogen lamps besides incandescent lamps for a brighter and cleaner future as well as saving on electricity bills^8,10,11^. The penetration of LED lamps varies across many markets and their sales are usually lower for lamp replacements in existing residential, public and commercial buildings than for installations in newly built buildings^8^. Country lighting assessments by the United Nations Environment Programme/Global Environment Facility (UNEP-GEF) en.lighten initiative indicated that a transition to efficient lighting globally would reduce 1044 terawatts-hours (TWh) of electricity (about 37% of global electricity use annually for lighting), saving over $120 billion in avoided electricity bills and reduce CO_2_ emissions by over 530 megatonnes (Mt) annually^7^.

Although the potential benefits of shifting to energy-efficient LED lighting in buildings located in different parts of the world have been demonstrated by some studies^12–16^, they have different results and insufficient comparable criteria, making it difficult to standardise the lighting energy consumption of the buildings for benchmarking. A typical energy consumption benchmarking tool for assessing a building’s overall energy performance is the building energy intensity (BEI). However, this parameter does not provide any information on the energy performance for lighting in the building. Also, there have been very few literatures reported on the lighting energy performance, its associated CO_2_ emissions and electricity costs for commercial buildings in the Southeast Asia region. This could be due to limitations in accessing the buildings and laborious data collection/measurements at the sites.

The aim of this work is to develop an estimation approach for evaluating the energy performance, CO_2_ emissions and electricity costs for lighting in commercial buildings in Southeast Asia that could assist the government and consumers in making decisions to transition to energy-efficient lighting. From the calculated data, we create a decarbonisation pathway for lighting in commercial buildings in the region. We apply the approach to a selected library in Brunei Darussalam to assess its energy consumption, CO_2_ emissions and electricity costs for lighting and we benchmarked it against the library building in Southeast Asia. Finally, we identify the best lighting system for the library. To the best of our knowledge, this work is the first to propose a generic estimation of lighting energy consumption, the associated CO_2_ emissions and electricity costs for commercial buildings in Southeast Asia and its decarbonisation pathway for benchmarking.

## Methods

### Data collection

Energy audit and lighting data of commercial buildings (such as hospital, hotel, library, mosque, office, retail and university) in Brunei Darussalam^17^, Malaysia^15,17–20^, Singapore^17,21^ and Thailand^22,23^ found in the literature were collected. The collected information include types of building, year of the energy audit conducted, annual electricity consumption, building floor area, building energy intensity and percentage of lighting usage. The data for a library building in Brunei Darussalam was collected and measured at the site. The library building and lighting information include building floor area, building operating hours, types of lamps installed and to be retrofitted, number of lamps installed, functional and to be retrofitted, and power rating of the lamp installed and to be retrofitted. Carbon intensities of the selected countries from 2005 to 2020 for CO_2_ emissions calculation were sourced from Our World in Data (https://ourworldindata.org/co2/country/brunei, accessed on June 13, 2022).

The electrical tariffs for commercial buildings in the selected countries were sourced from Department of Electrical Services, Brunei Darussalam (http://www.electrical.gov.bn/elektrik/SitePages/Tarif%20Elektrik.aspx, accessed on June 13, 2022), Tenaga Nasional Berhad, Malaysia (https://www.tnb.com.my/assets/files/Tariff_booklet.pdf, accessed on June 13, 2022), Energy Market Authority of Singapore (https://www.ema.gov.sg/statistic.aspx?sta_sid=20190319gX6WhmRypOKm, accessed on June 13, 2022) and Thailand Board of Investment (https://www.boi.go.th/index.php?page=utility_costs, accessed on June 13, 2022). The averaged electrical tariffs for commercial buildings in the respective countries were used to estimate the electricity costs of lighting, which are B$0.10/kWh for Brunei Darussalam, RM0.23/kWh (~ B$0.07/kWh) for Malaysia, S$0.26/kWh (~ B$0.26/kWh) for Singapore and Baht 3.32/kWh (~ B$0.13/kWh) for Thailand.

### Estimation of energy consumption, CO_2_ emissions and electricity costs for lighting

The energy consumption, CO_2_ emissions and electricity costs of lighting for each building type in the selected countries were calculated using the following equations:

1 $$\mathrm{Building \,energy\, intensity},\, BEI\, ({{\mathrm{kWh}}/{\mathrm{m}^{2}}}/{\mathrm{year}}):\,\,{ B}EI=\frac{E}{A}$$

2 $$\mathrm{Energy\, consumption\, for\, lighting}, \, {\overline{E} }_{L}\, (\mathrm{kWh}/\mathrm{m}^{2}/\mathrm{year}):\, \, { \overline{E} }_{L}=BEI\times L$$

3 $$\mathrm{Or}, \, \, {\overline{E} }_{L}=\frac{{E}_{L}}{A}$$

4 $${\mathrm{CO}}_{2}\, \mathrm{ emissions \, due \, to \, lighting},\, { EM}_{{CO}_{2}}\,({\mathrm{kg}\,{\mathrm{CO}_{2}}/{\mathrm{m}^{2}}}/{\mathrm{year}}):\, \, {EM}_{{CO}_{2}}={\overline{E} }_{L}\times CI$$

5 $$\mathrm{ Electricity\, cost\, for\, lighting},\, \, {C}_{L}\, (\mathrm{B\$}/\mathrm{m}^{2}/\mathrm{year}):\, \, {C}_{L} ={\overline{E} }_{L}\times ET$$

where $$E$$ is the annual energy consumption of the building (kWh/year), $${E}_{L}$$ is the annual energy consumption of lighting (kWh/year), $$A$$ is the floor area of the building (m^2^), $$L$$ is the percentage of averaged energy consumption of lighting for commercial buildings (%), $$CI$$ is the carbon intensity in the corresponding year (kg CO_2_/kWh) and $$ET$$ is the electrical tariff for commercial buildings (B$/kWh).

Linear regression models for energy consumption, CO_2_ emissions and electricity costs for lighting in commercial buildings in Southeast Asia were developed from the calculated data. The results were illustrated as correlation plots of energy consumption, CO_2_ emissions and electricity costs for lighting against BEI of commercial buildings in Southeast Asia. Then, a decarbonisation pathway for lighting in commercial buildings in Southeast Asia was proposed by creating a plot of CO_2_ emissions due to lighting against the corresponding year. The annual electricity cost of lighting for different types of commercial buildings in Southeast Asia was projected up to 2050, assuming an annual electricity price increase of 5%. The study also projected the annual electricity cost of lighting up to 2050 for commercial buildings in the same region for 5%, 10% and 15% increments in annual electricity price.

### Estimation of energy consumption, CO_2_ emissions and electricity costs of different lighting systems for a library building in Brunei Darussalam

The annual energy consumption of artificial (fluorescent and incandescent) and LED lighting, ${\overline{E} }_{L}$ (kWh/m^2^/year), for a library in Brunei Darussalam was calculated using Eq. (6):

6 $${\overline{E} }_{L}=\frac{H\times \sum {\left(N\times P\right)}_{i}}{A}$$

where $H$ is the building operating hours for the corresponding year (h), $N$ is the number of lamps, $P$ is the power rating of lamp (kW) (fluorescent lamp: 0.036 kW; incandescent lamp: 0.017 kW; LED T8 tube: 0.008 kW and LED bulb: 0.004 kW), subscript $i$ represents the types of lamps (fluorescent lamp, incandescent lamp, LED T8 tube or LED bulb) and $A$ is the floor area of the building (m^2^) (~ 1642 m^2^).

The library building in Brunei Darussalam considered in this study has a total of 423 lamps installed, of which 407 are fluorescent lamps and 16 are incandescent lamps. These quantities of lamps were assumed for 2010 when it is a new building. A site visit to the library in 2020 found that there was a total of 338 functional lamps, of which 326 are fluorescent lamps and 12 are incandescent lamps. For LED lighting energy consumption calculation, it was assumed that the same number of lamps were replaced such that the library building has 407 LED T8 tubes and 16 LED bulbs in 2010 and 338 LED T8 tubes and 12 LED bulbs in 2020. The annual energy consumption of artificial (fluorescent and incandescent) and LED lighting were projected for 2030, 2040 and 2050, assuming that they have the same percentage decrease as that between 2010 and 2020 (i.e., 20.7%). For artificial and natural (or mixed) lighting energy consumption calculation, a 14%^24^ energy saving from artificial lighting in the library building was accounted for in 2010, 2020, 2030, 2040 and 2050.

Then, the calculated annual energy consumption for different lighting systems were applied to the derived correlation shown in Fig. 2a to obtain the corresponding BEI values for the library with different lighting systems. With known BEI, the annual CO_2_ emissions of different lighting systems can be estimated using the derived correlation shown in Fig. 2b. The energy consumption and decarbonisation pathway of different lighting systems for a library in Brunei Darussalam were compared with commercial buildings in the region. The annual electricity costs of different lighting systems for the library building were calculated using Eq. (5) for 2010 and projected up to 2050, assuming an annual electricity price increase of 5%. The result was compared with the annual electricity cost of lighting for a library in the region. Lastly, the calculated and estimated CO_2_ emissions and annual electricity cost of lighting for a library building in Brunei Darussalam were compared to evaluate the accuracy of the correlations proposed in this study.

## Results and discussion

### Energy consumption of lighting for commercial buildings

**Figure 1:**
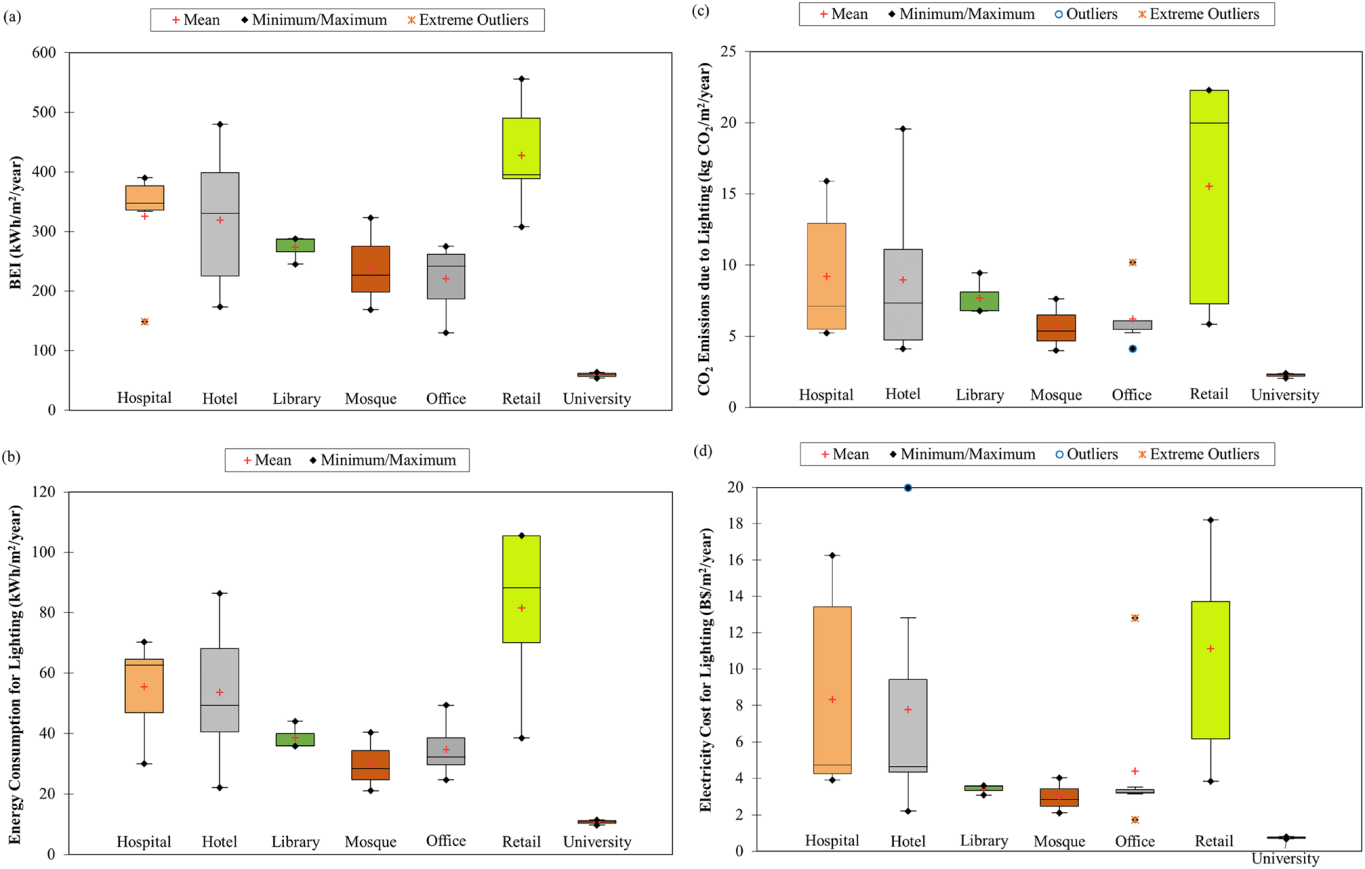
Box plots of **(a)** building energy intensity (BEI), **(b)** energy consumption, **(c)** CO_2_ emissions and **(d)** electricity cost of lighting for different types of commercial buildings in Southeast Asia.

The collected energy and lighting data for commercial buildings (such as hospital, hotel, library, mosque, office, retail and university) in Brunei Darussalam, Malaysia, Singapore and Thailand were used to calculate the BEI and percentage of energy consumption of lighting for each building type in the respective countries. Results showed that commercial buildings from these Southeast Asia countries have BEI ranging from 54.09 kWh/m^2^/year to 556 kWh/m^2^/year with a mean value of 281.85 kWh/m^2^/year (Table 1). The box plot in Fig. 1a shows that retail building consumes the most energy (mean = 427.60 kWh/m^2^/year) among the assessed buildings, indicating high energy-consuming activities (such as lighting, heating, cooling, ventilation and electrical appliances) in the building.

**Table 1:** Descriptive statistics of building energy intensity (BEI), energy consumption, CO_2_ emissions and electricity cost of lighting for commercial buildings in Southeast Asia.

Statistic	BEI (kWh/m^2^/year)	Energy consumption of lighting (kWh/m^2^/year)	CO_2_ emissions due to lighting (kg CO_2_/m^2^/year)	Electricity cost of lighting (B$/m^2^/year)
Minimum	54.09	9.74	2.06	0.68
Maximum	556.00	105.50	22.29	19.98
Mean	281.85	46.94	8.41	6.25
Standard deviation	123.45	25.24	5.64	5.48

Our study found that, on average, the percentage of energy consumption of lighting for commercial buildings in Southeast Asia was about 17.1% (12.5% for Brunei Darussalam^17^, 18.0% for Malaysia and Singapore^25^, and 19.9% for Thailand). These values can be used to determine the energy consumption of lighting for the building, which has been very few being reported in the literature due to the laborious field measurements involved. We calculated that the mean energy consumption of lighting for commercial buildings in Southeast Asia was 46.94 kWh/m^2^/year with a minimum value of 9.74 kWh/m^2^/year and a maximum value of 105.50 kWh/m^2^/year (Table 1). Among the assessed countries, commercial buildings in Brunei Darussalam seem to consume the least energy for lighting (mean = 33.94 kWh/m^2^/year) (Table 2). The box plot in Fig. 1b shows that retail building has the highest lighting energy consumption (mean = 81.54 kWh/m^2^/year) among the assessed buildings and this could be due to the high usage of less energy-efficient lamps in the building.

**Table 2:** Energy consumption, CO_2_ emissions and electricity cost of lighting for commercial buildings in Southeast Asia.

Building Type	Energy consumption for lighting (kWh/m^2^/year)					CO_2_ emissions due to lighting (kg CO_2_/m^2^/year)					Electricity cost for lighting (B$/m^2^/year)				
	Brunei Darussalam	Malaysia	Singapore	Thailand	Mean	Brunei Darussalam	Malaysia	Singapore	Thailand	Mean	Brunei Darussalam	Malaysia	Singapore	Thailand	Mean
Hospital	41.75	67.74	62.55	30.00	55.39	7.89	15.25	5.22	6.34	9.19	4.18	4.74	16.26	3.90	8.35
Hotel	34.25	72.90	63.09	34.70	53.60	6.47	16.50	4.70	7.33	8.96	3.43	5.10	16.40	4.51	7.77
Library	35.92	44.05	–	–	38.63	6.79	9.44	–	–	7.67	3.59	3.08	–	–	3.42
Mosque	29.96	–	–	–	29.96	5.66	–	–	–	5.66	3.00	–	–	–	3.00
Office	32.25	34.85	49.32	27.10	34.70	6.09	7.71	4.11	5.73	6.22	3.23	2.44	12.82	3.52	4.41
Retail	38.50	88.20	70.02	105.50	81.54	7.27	19.97	5.84	22.29	15.53	3.85	6.17	18.21	13.72	11.13
University	–	10.66	–	–	10.66	–	2.25	–	–	2.25	–	0.75	–	–	0.75
Mean	33.94	46.84	61.77	60.56	46.94	6.41	10.46	4.97	12.80	8.41	3.93	3.28	16.06	7.87	6.25

**Figure 2:**
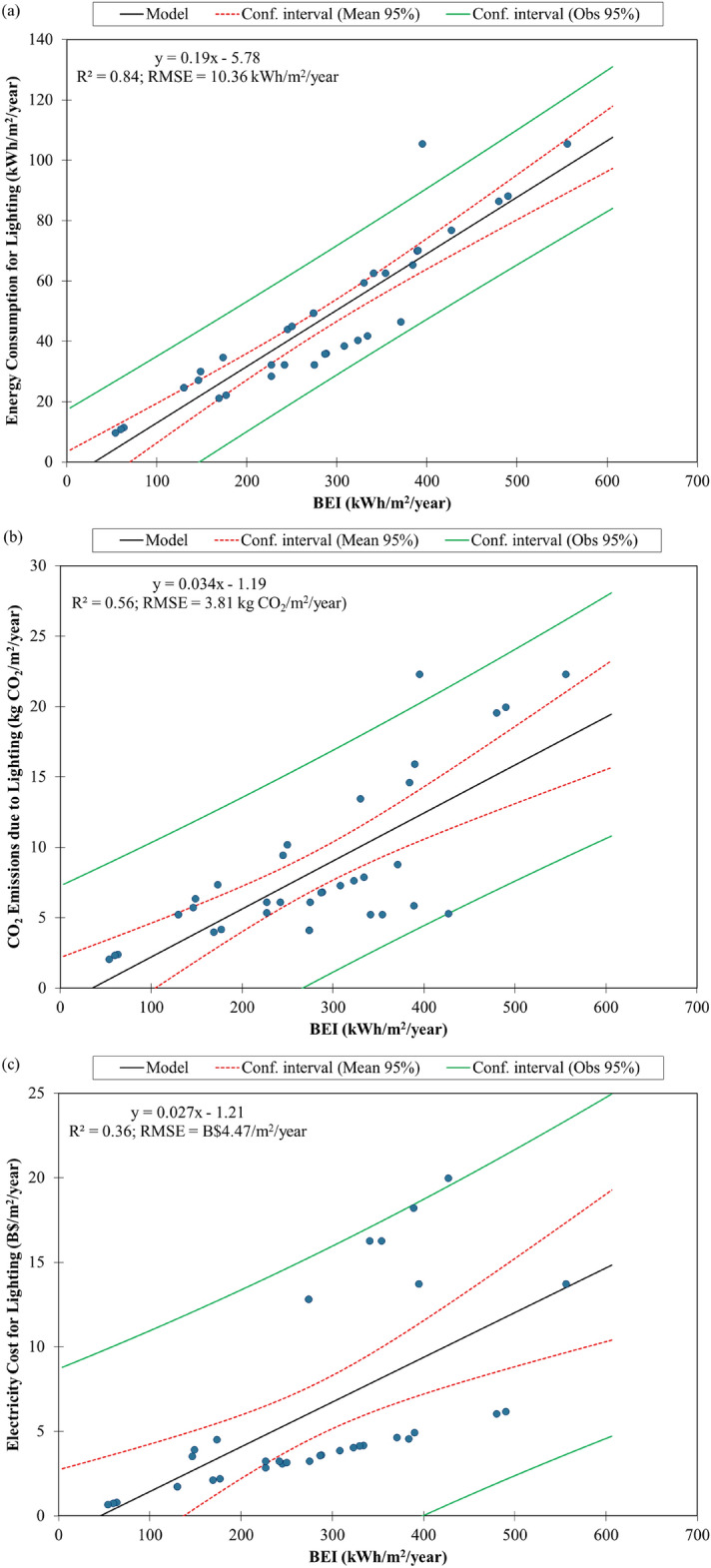
Correlation plots between building energy intensity (BEI) and **(a)** energy consumption, **(b)** CO_2_ emissions and **(c)** electricity costs for lighting by linear regression model for commercial buildings in Southeast Asia with 95% confidence interval.

**Figure 3:**
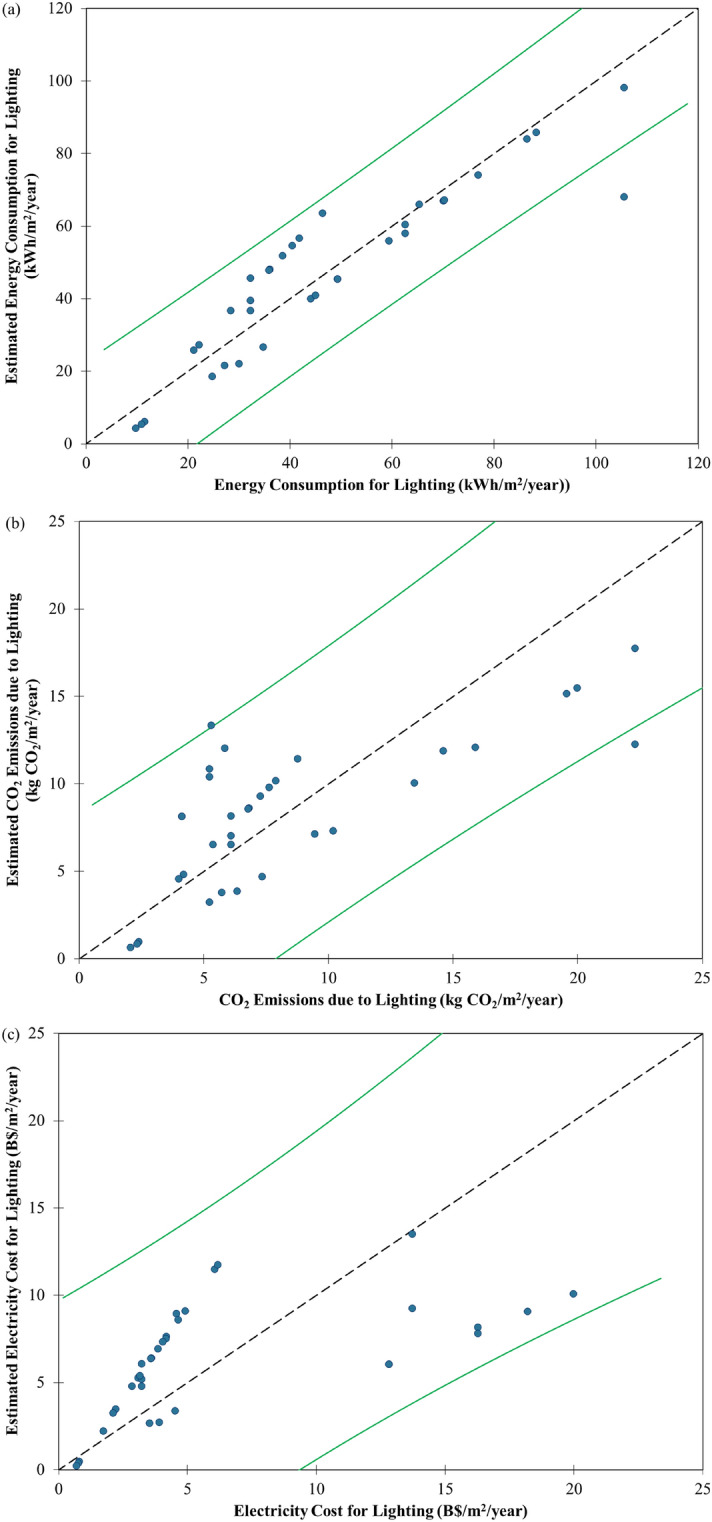
Scatter plots of calculated and estimated **(a)** energy consumption, **(b)** CO_2_ emissions and **(c)** electricity costs for lighting by linear regression model for commercial buildings in Southeast Asia with 95% confidence interval (represented by the green lines).

A linear regression model for lighting energy consumption for commercial buildings in Southeast Asia was developed from a correlation plot between BEI and lighting energy consumption (Fig. 2a). It can be used to estimate the lighting energy consumption for any commercial buildings in the region provided that the BEI of the building is known. We expect commercial buildings with a high BEI value would consume more energy for lighting than those with a lower BEI value. For example, a commercial building with a BEI of 600 kWh/m^2^/year was estimated to consume 108.22 kWh/m^2^/year while that with a BEI of 100 kWh/m^2^/year was estimated to consume 13.22 kWh/m^2^/year. The model seems to have a good fitting to the calculated data, as indicated by its high ${R}^{2}$ value of 0.84 and its low root mean square error (RMSE) of 10.36 kWh/m^2^/year. Many data points were observed within the narrow mean 95% confidence interval of the model (Fig. 2a). The most accurate estimates of lighting energy consumption by the model seem to be for commercial buildings with BEI values between 200 kWh/m^2^/year and 400 kWh/m^2^/year. The comparison between calculated and estimated lighting energy consumption in Fig. 3a shows that the model has satisfactory estimation accuracy for lighting energy consumption since there are many data points observed near the line of equality (i.e., the y = x line) and within the 95% confidence interval.

### CO_2_ emissions and decarbonisation pathway for lighting in commercial buildings

We found that the associated CO_2_ emissions for lighting in commercial buildings in the selected countries ranged between 2.06 kg CO_2_/m^2^/year and 22.29 kg CO_2_/m^2^/year with a mean value of 8.41 kg CO_2_/m^2^/year (Table 1). The highest CO_2_ emissions due to lighting were produced from the retail building (mean = 15.53 kg CO_2_/m^2^/year) (Fig. 1c) since it consumes the most energy for lighting than other types of building assessed in this study. Although commercial buildings in Brunei Darussalam have the lowest energy consumption for lighting, their associated CO_2_ emissions (mean = 6.41 kg CO_2_/m^2^/year) were about 1.3 times higher than those in Singapore (mean = 4.97 kg CO_2_/m^2^/year), the lowest emitter of CO_2_ from lighting usage among the assessed Southeast Asian countries (Table 2). This was because Brunei Darussalam has a higher carbon intensity with a mean value of 0.18 kg CO_2_/m^2^/year (from 2000 to 2019) than Singapore, which has a mean value of 0.07 kg CO_2_/m^2^/year (from 2000 to 2020). The highest emitter of CO_2_ from lighting usage among the assessed countries was Thailand (mean = 12.80 kg CO_2_/m^2^/year) (Table 2) due to its high carbon intensity and energy consumption for lighting.

**Figure 4:**
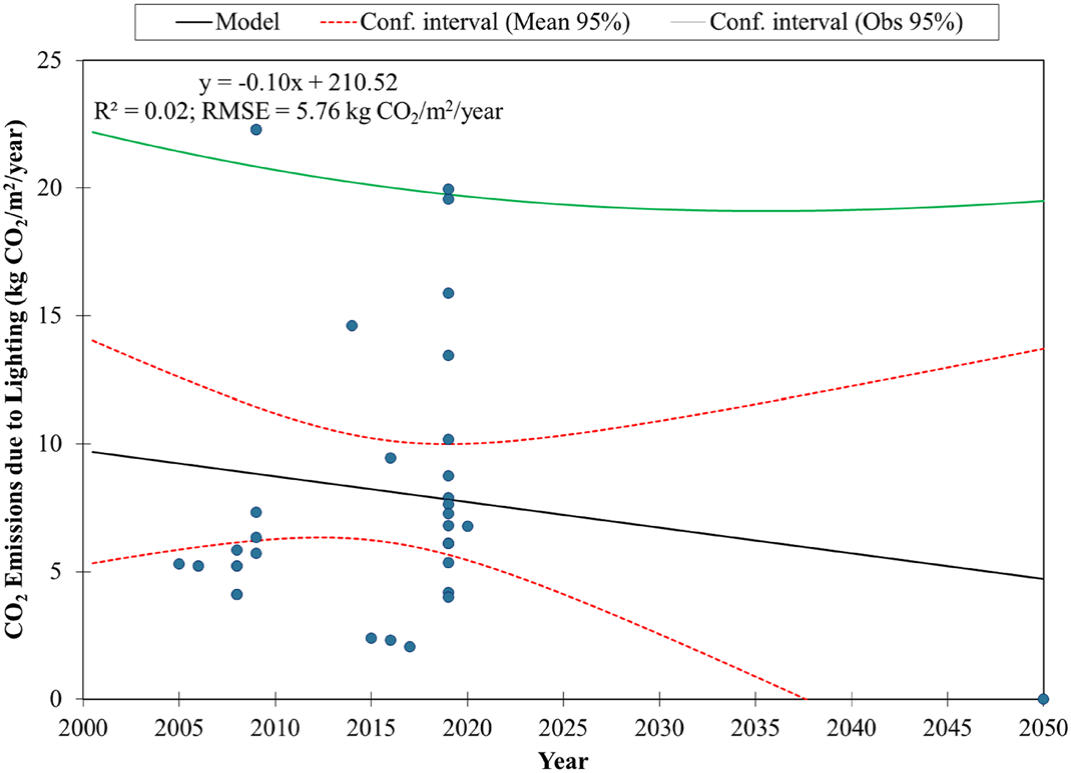
Decarbonisation pathway for lighting by linear regression model for commercial buildings in Southeast Asia with 95% confidence interval.

The correlation plot between BEI and CO_2_ emissions due to lighting for commercial buildings in Southeast Asia is shown in Fig. 2b. The linear regression model derived from these calculated data can be used to estimate the CO_2_ emissions due to lighting for any commercial buildings in the region with a known BEI value. We estimated that a commercial building with a BEI of 600 kWh/m^2^/year would emit 19.21 kg CO_2_/m^2^/year while that with a BEI of 100 kWh/m^2^/year would emit 2.21 kg CO_2_/m^2^/year. This model has a moderate ${R}^{2}$ value of 0.56 and a low RMSE value of 3.81 kg CO_2_/m^2^/year. Hence, there are lesser data points being observed within the narrow mean 95% confidence interval than the observational 95% confidence interval (Fig. 2b). The model seems to give the most accurate estimation of CO_2_ emissions due to lighting for commercial buildings with BEI values between 200 kWh/m^2^/year and 320 kWh/m^2^/year. The estimated CO_2_ emissions due to lighting can be considered accurate since almost all the data points were observed within the 95% confidence interval (Fig. 3b) despite less data points near the line of equality. Then, a decarbonisation pathway for lighting in commercial buildings in Southeast Asia up to 2050 was developed by linear regression of the calculated CO_2_ emissions data against the corresponding year (Fig. 4). It can be used as a benchmark for commercial buildings in the region. A green building would have a decarbonisation pathway below the benchmark for at least the next two decades.

Life cycle assessment (LCA) could also be used to assess the potential environmental impacts of lighting systems (using metrics such as the global warming potential or the CO_2_ emissions), its materials and energy resources over its whole life cycle. Normally, the LCA study includes environmental information such as CO_2_ emissions, acidification, eutrophication, energy, waste, water and recycled content^26^. Although LCA methodology gives comprehensive information, it is more complex and difficult to compare between different studies on lighting systems (due to limitations in methodology and data availability, leading to different assumptions being made^27^) than the approach presented in this work.

### Electricity costs of lighting for commercial buildings

The electricity costs associated with lighting for commercial buildings in the selected countries were calculated and they ranged from B$0.68/m^2^/year to B$19.98/m^2^/year with a mean value of B$6.25/m^2^/year (Table 1). Of the building types considered in this study, the retail building has the highest electricity cost for lighting (B$11.13/m^2^/year) due to its high energy consumption for lighting (Fig. 1d). Since the electrical tariff for commercial buildings in Brunei Darussalam is more expensive than in Malaysia, the electricity cost of lighting for commercial buildings in Brunei Darussalam (mean = B$3.93/m^2^/year) was about 1.2 times higher than in Malaysia (mean = B$3.28/m^2^/year) (Table 2) despite having lower lighting energy consumption and CO_2_ emissions. We found that the electricity cost of lighting for commercial buildings among the assessed countries was the most expensive in Singapore with a mean value of B$16.06/m^2^/year (Table 2) due to its high electrical tariff.

Figure 2c shows the correlation plot between BEI and electricity cost of lighting for commercial buildings in Southeast Asia. From the derived linear regression model, we estimated that the electricity cost of lighting for a commercial with a BEI of 600 kWh/m^2^/year would be B$14.99/m^2^/year while that with a BEI of 100 kWh/m^2^/year would be B$1.49/m^2^/year. Since many data points were observed beyond the mean 95% confidence interval, the model has a low $${R}^{2}$$ value of 0.36 and a moderate RMSE value of B$4.47/m^2^/year (Fig. 2c). The model seems to give the most accurate estimation of electricity cost of lighting for commercial buildings with BEI values between 220 kWh/m^2^/year and 360 kWh/m^2^/year. Although there are not many data points observed near the line of equality, the estimated electricity cost of lighting can be considered accurate since they all lie within the 95% confidence interval (Fig. 3c).

**Figure 5:**
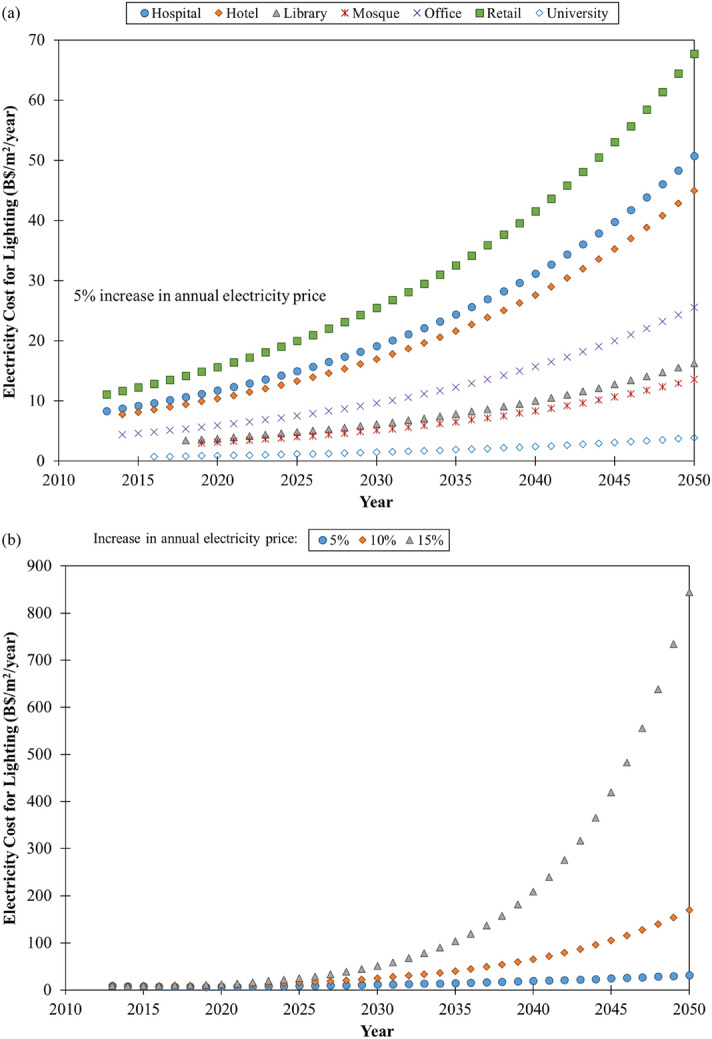
Projection of electrical costs of lighting for **(a)** different types of commercial buildings in Southeast Asia with a 5% increase in annual electricity price and **(b)** commercial buildings in the same region with 5%, 10% and 15% increase in annual electricity price.

The electricity costs of lighting for different types of commercial buildings in Southeast Asia were projected up to 2050 with a 5% increase in annual electricity price (Fig. 5a). Our results show that the electricity costs for lighting would increase to B$3.92/m^2^/year (for university buildings) and it can reach as high as B$67.70/m^2^/year (for retail buildings) by 2050 if there is no major reduction in lighting energy consumption since the last decade. We also projected the electricity costs of lighting for commercial buildings in the region with 5%, 10% and 15% increase in annual electricity price (Fig. 5b) to demonstrate the effect of increasing the annual electricity price. A significant rise of about 26.5 times in electricity costs for lighting can be expected when the annual electricity price was increased from 5 to 15% in 2050.

### Application to a library building in Brunei Darussalam

**Figure 6:**
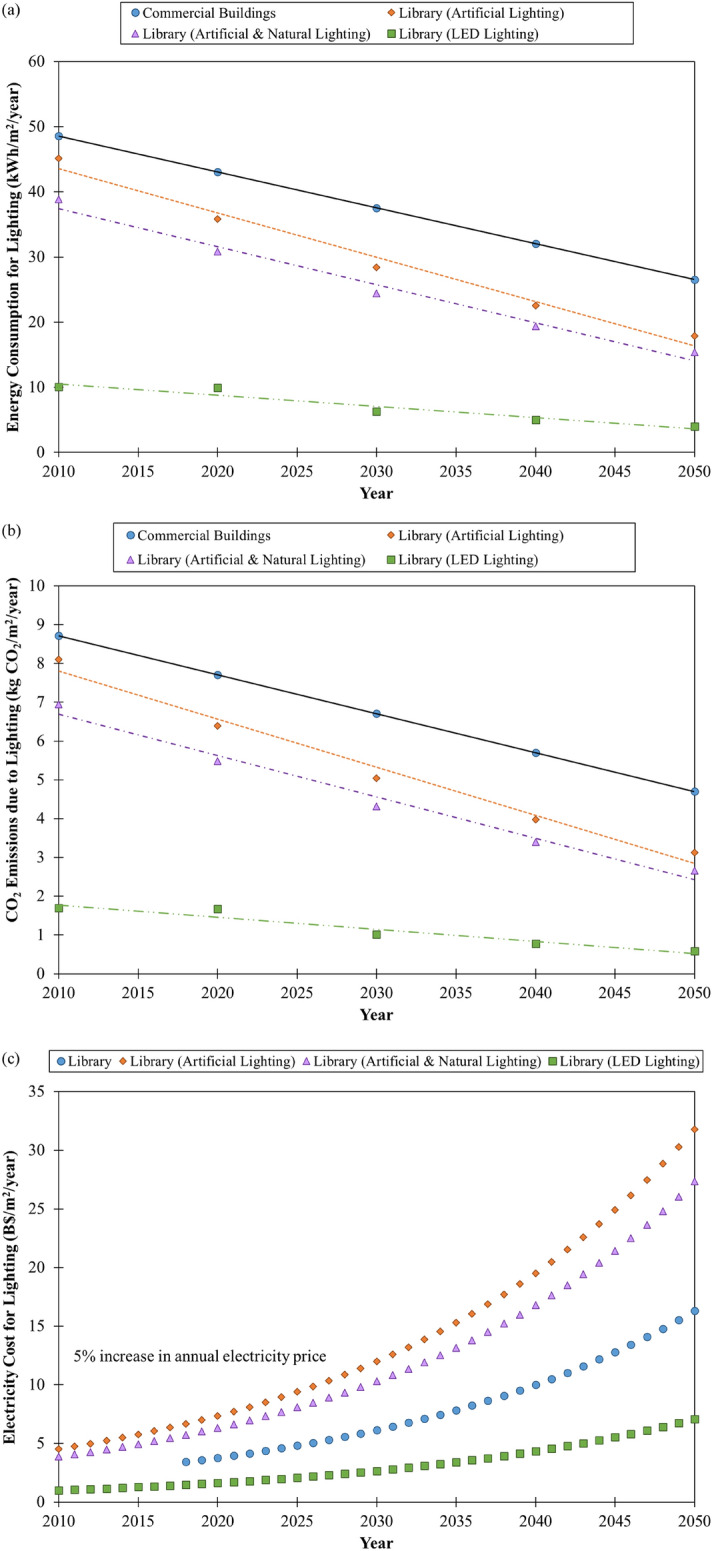
**(a)** Estimated energy consumption, **(b)** decarbonisation pathway and **(c)** projected electricity costs (with a 5% increase in annual electricity price) of different lighting systems for a library building in Brunei Darussalam in comparison to commercial buildings in Southeast Asia.

We calculated and projected the lighting energy consumption for a selected library building in Brunei Darussalam with three different lighting systems, namely: (1) artificial lighting that consists of fluorescent and incandescent lamps, (2) artificial and natural (or mixed) lighting, and (3) LED lighting up to 2050 to determine the most sustainable lighting system for greener building. As seen in Fig. 6a, the lighting energy consumption of the assessed library building with artificial lighting (17.88 kWh/m^2^/year) was expected to be about 1.5 times lesser than the commercial buildings in Southeast Asia (26.53 kWh/m^2^/year) by 2050. The energy consumption for the library can be slightly reduced to about 1.7 times by adopting a mixed lighting system (15.37 kWh/m^2^/year) as a result of less lamp usage and it can be reduced further to about 6.7 times by retrofitting all the existing lamps with LED lamps (3.98 kWh/m^2^/year) by 2050 when comparing them with the commercial buildings in Southeast Asia. This shows that LED lamps are more energy-efficient than fluorescent and incandescent lamps. Further energy savings are possible by deploying advanced lighting controls such as occupancy sensors, scheduling, multi-level lighting/dimming, building automation system, demand-responsive lighting, daylight harvesting, plug-load control and high-end trimming/light-level tuning in buildings^28–30^ in addition to the LED lamps. Williams et al. estimated that the average lighting energy savings potential from lighting controls in commercial buildings were 24% for occupancy strategies, 28% for daylighting strategies, 31% for personal tuning, 36% for institutional tuning and 38% for multiple approaches^30^.

We applied the calculated energy consumption for different lighting systems for the selected library building in Brunei Darussalam to the derived models to determine their CO_2_ emissions due to lighting that would enable us to develop the decarbonisation pathways for the assessed library with the respective lighting systems. The results in Fig. 6b show that the assessed library is considered a green building in terms of lighting because its CO_2_ emissions due to lighting were below those for commercial buildings in Southeast Asia. Since energy consumption is proportional to CO_2_ emissions, the same trend was observed for all the investigated lighting systems for the library. The CO_2_ emissions due to lighting could be significantly reduced to 0.59 kg CO_2_/m^2^/year if the library building upgrade its lighting system to LED lighting, which was about 8 times lesser than the CO_2_ emissions due to lighting for commercial buildings in Southeast Asia (4.70 kg CO_2_/m^2^/year) by 2050. Zhang et al. conducted an LCA study on five United States (U.S.) Department of Energy (DOE) prototypical commercial buildings consisting of a small office, medium office, sit-down restaurant, standalone retail and primary school in the U.S^31^. Their study showed that incandescent lights emit less CO_2_ per lamp but their long-term CO_2_ emissions were the highest compared to the compact fluorescent lamp (CFL) and LED due to short lifetime and more bulb replacements. The LCA study by Bertin et al. on lighting systems for indoor workplaces in France indicated that enhancing the LED lamps’ lifetime and manufacturing processes would bring a greater reduction in potential environmental impacts for both lamp replacement and new buildings/refurbishment^27^.

As seen in Fig. 6c, the projected electricity costs of lighting for the assessed library in 2050 were nearly twice for an artificial lighting system (B$31.80/m^2^/year) and higher by about 1.7 times for a mixed lighting system (B$27.35/m^2^/year) when compared to the library building in Southeast Asia (B$16.31/m^2^/year). This indicates that the library requires technical action to reduce its electricity cost for lighting. The results show that an LED lighting system is the best option for the assessed library to greatly reduce its electricity costs for lighting to B$7.07/m^2^/year, which was about 18.7 times below the electricity costs of lighting benchmarked for a library building in Southeast Asia.

## Conclusions

The present work demonstrates an estimation approach based on linear regression models that could be used to approximate the energy consumption, CO_2_ emissions and electricity costs of lighting for commercial buildings in Southeast Asia. This could be a powerful and quick tool for assessing the energy, environmental and economic aspects of lighting in commercial buildings. We developed a decarbonisation pathway for lighting in commercial buildings in Southeast Asia from the calculated data. The derived models have been applied to a library building in Brunei Darussalam and the results indicate that the building requires technical action to reduce its electrical costs for lighting although it meets the decarbonisation pathway for lighting in commercial buildings in the region. We show that a lighting upgrade to LED lighting could significantly reduce the lighting energy consumption, its associated CO_2_ emissions and electricity costs, thus making the library building greener. Further research can include developing different statistical models for estimating the energy consumption, CO_2_ emissions and electricity costs for lighting, applications of the work to other building sectors (for examples, residential and industrial buildings) and comparing the presented approach with a real case study in which the lighting energy consumption is measured before and after artificial lighting renovation to quantify the error of the estimation method.

## Data Availability

All the relevant data are included in the published article.
